# Meetings

**Published:** 1891-12

**Authors:** 


### Bristol Medico-Chirurgical Society.

*Annual Meeting, October 14th,* 1891.

Mr. S. H. Swayne, President, in the Chair.

After a vote of thanks to the retiring President (Mr. S. H. Swayne) had been unanimously passed, his successor, Mr. F. R. Cross, took the Chair and read his inaugural address (see page 241), for which a cordial vote of thanks was given hlm.

The Honorary Secretary, Assistant-Editor and Honorary Librarian read their annual reports, which were adopted, and officers for the ensuing session were elected. Dr. Markham Skerritt was chosen President-elect.

Dr. Watson Williams read notes on (1) a case of laryngeal tuberculosis of the proliferative type, and on (2) a case of choreic spasm of the occipito-frontalis.

Mr. Paul Bush exhibited (1) a large triangular piece of crockery which had caused a rapidly fatal perforation of the gluteal artery, and (2) an enormously hypertrophied labium which he had removed from a patient.

---

*November 11th,* 1891.

Mr. F. R. Cross, President, in the Chair.

Mr. F. P. Lansdown exhibited a patient who had been cured of a large femoral aneurism by digital compression applied continuously for forty-eight hours.

Dr. Ewen Maclean showed a marked case of hæmophilia in a boy. The disease came on suddenly. No cause could be assigned for the condition.

Dr. Clements Hailes read a paper on Corneal Ulcers, and exhibited several patients with various forms of this affection. He commented on the position of the ulcers and their causes, both constitutional and local. Amongst the former were foreign bodies, burns, irritating discharges, injuries and diseases of the lids, paralysis of 7th nerve, &c. Amongst the constitutional conditions were struma, under-feeding and bad hygienic surroundings. As modes of treatment were mentioned the actual cautery, paracentesis of the anterior chamber, sulphur, &c.

Mr. Nelson C. Dobson exhibited a papillomatous growth which he had removed from the broad ligament of a patient aged 19, who had suffered from pain and enlargement of abdomen, &c. On the second day after the operation severe peritonitis supervened; the wound was re-opened and drained, and recovery took place. Mr. Dobson commented on the difficulty of diagnosis in such cases, and on the recovery from an apparently hopeless condition of peritonitis by re-opening the wound and draining.

Dr. Michell Clarke showed numerous naked-eye and microscopic specimens, illustrated by drawings, of three cases of cirrhosis of the liver, with jaundice. The first, in a child of seven months, appeared to be an acute diffuse cirrhosis with endarteritis and atrophy of the hepatic cells. In the second case, that of a boy aged 12, there was biliary cirrhosis, intracellular and perilobular. The third case was ordinary atrophic cirrhosis with long-continued jaundice, but no ascites. Dr. Clarke made some remarks on the cause of death in such cases and in long-continued jaundice.

---

*December 9th*, 1891.

Mr. F. R. Cross, President, in the Chair.

Mr. C. A. Morton exhibited two patients with congenital dislocation of the hip: in one case, a boy, bilateral; in the other, a girl, unilateral. Lordosis was very marked in the former; in the latter the limb was *everted.*

Mr. A. W. Prichard read notes on a case of anthrax in a tanner. The disease appeared on the finger; there was a marked slough, and the appearance was that of a bad whitlow. Bacilli were found in the discharge from the wound. The affected part was excised and treated with zinc chloride. Recovery followed.

Mr. Bush also described a case of anthrax which was contracted by a man whilst cutting up a diseased cow. There was a black central slough on the hand. The spleen of the diseased animal was eaten by a sow with a litter of young. The sow and several of her offspring were affected and died of the disease. Free excision of the part was followed by recovery.

Dr. W. J. Fyffe read a paper on an outbreak of Rötheln. (This paper will be published *in extenso.*)

Dr. Edgeworth read an abstract of a paper on an Anatomical Investigation into some of the Sources of Nerve-supply to the Viscera. Certain large fibres had been found which he considered to be sensory, (1) because of their connection with Pacinian corpuscles, and (2) because by a series of continuous sections they could be traced up to the posterior roots. Dr. Edgeworth thought the presence of these fibres might help to explain "referred visceral pains," as in angina pectoris, stone in the kidney, &c.

G. Munro Smith, *Hon. Sec.*

